# Utility of Multi-Modal MRI for Differentiating of Parkinson's Disease and Progressive Supranuclear Palsy Using Machine Learning

**DOI:** 10.3389/fneur.2021.648548

**Published:** 2021-04-14

**Authors:** Aron S. Talai, Jan Sedlacik, Kai Boelmans, Nils D. Forkert

**Affiliations:** ^1^Department of Radiology, University of Calgary, Calgary, AB, Canada; ^2^Department of Diagnostic and Interventional Neuroradiology, University Medical Center Hamburg-Eppendorf, Hamburg, Germany; ^3^Department of Neurology, University Hospital Würzburg, Würzburg, Germany; ^4^Department of Neurology, Klinikum Bremerhaven-Reinkenheide, Bremerhaven, Germany

**Keywords:** machine learning, magnetic resonance imaging, computer-assisted image analysis, Parkinson's disease, progressive supranuclear palsy

## Abstract

**Background:** Patients with Parkinson's disease (PD) and progressive supranuclear palsy Richardson's syndrome (PSP-RS) often show overlapping clinical features, leading to misdiagnoses. The objective of this study was to investigate the feasibility and utility of using multi-modal MRI datasets for an automatic differentiation of PD patients, PSP-RS patients, and healthy control (HC) subjects.

**Material and Methods:** T1-weighted, T2-weighted, and diffusion-tensor (DTI) MRI datasets from 45 PD patients, 20 PSP-RS patients, and 38 HC subjects were available for this study. Using an atlas-based approach, regional values of brain morphology (T1-weighted), brain iron metabolism (T2-weighted), and microstructural integrity (DTI) were measured and employed for feature selection and subsequent classification using combinations of various established machine learning methods.

**Results:** The optimal machine learning model using regional morphology features only achieved a classification accuracy of 65% (67/103 correct classifications) differentiating PD patients, PSP-RS patients, and HC subjects. The optimal machine learning model using only quantitative T2 values performed slightly better and achieved an accuracy of 75.7% (78/103). The optimal classifier using DTI features alone performed considerably better with 95.1% accuracy (98/103). The optimal multi-modal classifier using all features also achieved an accuracy of 95.1% but required more features and achieved a slightly lower F1-score compared to the optimal model using DTI features alone.

**Conclusion:** Machine learning models using multi-modal MRI perform significantly better than uni-modal machine learning models using morphological parameters based on T1-weighted MRI datasets alone or brain iron metabolism markers based on T2-weighted MRI datasets alone. However, machine learnig models using regional brain microstructural integrity metrics computed from DTI datasets perform similar to the optimal multi-modal machine learning model. Thus, given the results from this study cohort, it appears that morphology and brain iron metabolism markers may not provide additional value for classification compared to using DTI metrics alone.

## Introduction

The early differential diagnosis of classical Parkinson's disease (PD) and progressive supranuclear palsy (PSP)—in particular the Richardson's syndrome (PSP-RS)—is often limited by overlapping symptom profiles, which are not effectively captured by existing clinical scores or established diagnostic methods. Within this context, failure rates of up to 24% have been reported, even by movement disorders specialists (1). In clinical practice, diagnosis of PD and PSP-RS is mostly based on clinical examination including key features, response to levodopa, and established scores such as the Unified PD Rating Scale (UPDRS) (2). However, due to significant overlap of clinical symptoms and inadequate accuracy of bedside tests, differential diagnosis is often challenging, particularly in the early disease course. The relevance of an accurate early diagnosis is closely related to better disease management via appropriate drug administration, patient care protocols, and might improve disease prognosis considerably. Furthermore, the identification of early disease manifestations may lead to better targeted pharmaceutical therapies and enable advancements in developing more effective drug therapies in this domain.

In this context, group-wise studies using various magnetic resonance imaging (MRI) modalities such as T1-weighted (3, 4), T2-weighted (5, 6), and diffusion-tensor MRI (DTI) (7, 8) have shown significant differences between PD and PSP-RS patients and healthy control (HC) subjects. These differences indicate alterations in regional brain volume, brain iron metabolism, and microstructural brain tissue degradation, all off which are closely related to the neurodegenerative profiles of PD and PSP-RS compared to HC subjects (9–11). Supervised machine learning techniques are capable of identifying complex patterns in high-dimensional data, whereas the identified patterns can then be used to make patient-specific predictions on new unseen cases (12). Machine learning has been used successfully for various precision medicine problems (13) and multiple studies have attempted to utilize features obtained from the aforementioned group-wise studies to classify individual PD and PSP-RS patients [e.g., (14–16)].

However, only a few scientific studies have truly tried to harness the power of multi-modal imaging features to improve differential classification of patients with PD and PSP-RS so far [e.g., (17, 18)]. Moreover, the true benefit of using multi-modal imaging over single modality imaging information has not been explored in detail yet. Therefore, this study aims to present a comprehensive end-to-end framework to classify patients with PD, patients with PSP-RS, and HC subjects using T1-weighted, T2-weighted, and DTI datasets and evaluate the accuracy of optimal machine learning models trained using individual single modality features as well as multi-modality features.

## Materials and Methods

### Patients and Imaging

The study cohort used for this work has been previously described in detail by Boelmans et al. (16). Briefly described, 45 PD, 20 PSP-RS, and 38 HC subjects presenting to the movement disorder outpatient clinic of the Neurology Department of the University Medical Center Hamburg-Eppendorf between July 2009 and September 2010 were used for this secondary, retrospective study. No sample size estimates were computed or required for the primary observational study. The clinical diagnosis of PD and PSP-RS was conducted according to the UK Brain Bank criteria (19) and the National Institute of Neurological Disorders and Stroke and Society for PSP (NINDS-SPSP) (20), respectively. The inclusion criteria for the PSP-RS group were probable PSP-RS subjects presenting as classical Richardson's syndrome with vertical palsy, axial rigidity, and balance instability with early falls. PSP-RS patients who exhibited prominent freezing phenomena, asymmetric clinical features, and a clinically relevant levodopa response were excluded from the study. The clinical characteristics of the study participants are summarized in Table 1. The study was approved by the local ethics committee and informed consent was attained from all subjects.

**Table 1:** Demographic and clinical characteristics of study participants.

	**Parkinson's disease**	**Progressive supranuclear palsy**	**Healthy controls**
Number of patients	45	20	38
Sex, M/F	33/12	9/11	24/14
Age at examination, y, mean ± SD (range)	66.1 ± 7.2 (45–77)	71.2 ± 5.7 (59–79)	61.9 ± 11.3 (41–80)
Disease duration, y, mean ± SD (range)	13.7 ± 6.6 (2–30)	6.1 ± 3.4 (1–12)	-
Hoehn&Yahr, mean ± SD (range)	2.6 ± 0.8 (1–4)	2.6 ± 0.8 (1–4)	-
UPDRS motor score (OFF condition), mean ± SD (range)	37.4 ± 13.1 (14–63)	32.8 ± 12.0 (9–52)	-
UPDRS motor score (ON condition), mean ± SD (range)	20.0 ± 10.7 (5–52)	28.9 ± 10.8 (6–48)	-
MMSE, mean ± SD (range)	28.1 ± 1.4 (23–30)	25.1 ± 2.8 (19–29)	-

**Figure 1:**
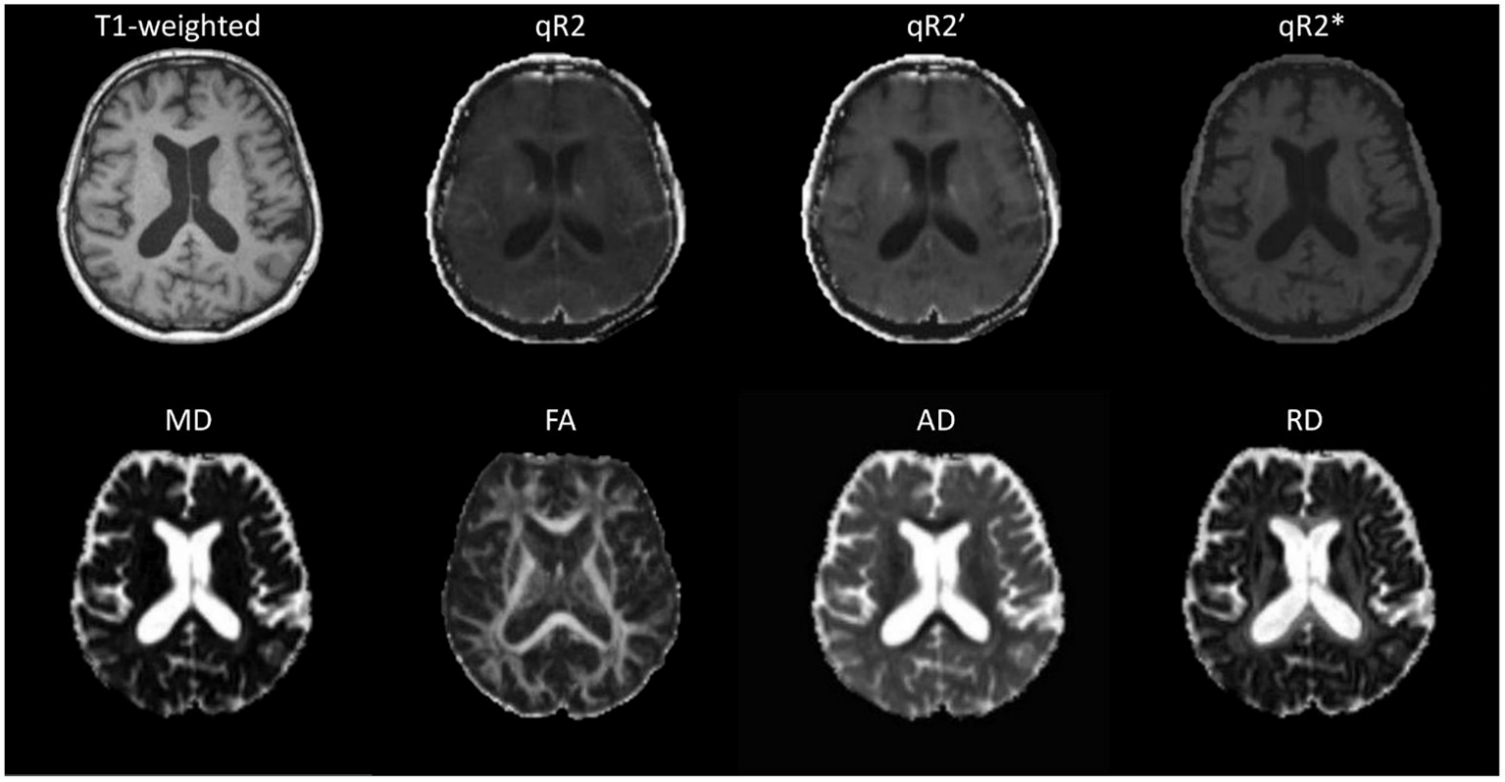
Selected slice from a multi-modal MRI dataset of a patient with Parkinson's disease.

All participants were scanned at the University Medical Center Hamburg-Eppendorf, Germany, using a 3T Siemens Skyra MR scanner (Figure 1). Among others, T1-weighted MPRAGE, DTI, and triple-echo T2- and T2^*^-weighted MRI datasets were acquired for each patient. The high-resolution T1-weighted MPRAGE dataset was acquired using TR = 1,900 ms, TE = 2.46 ms, flip angle = 90°, TI = 900 ms, image in-plane resolution of 0.94 ×0.94 mm^2^, and 0.94 mm slice thickness.

T2-weighted and T2^*^-weighted image sequences were acquired for this study for R2, R2^*^, and R2′ mapping. In detail, for R2 mapping, a spin-echo sequence with 15 echoes per shot was employed to record images using echo times of 12, 85, and 158 ms. A total of 24 slices with a thickness of 5 mm with a field-of-view of 240 mm were acquired using a repetition time of 4,590 ms and a flip angle of 150°. The T2^*^-weighted images were acquired with the same settings except for using a single shot echo-planar sequence with TEs of 21, 52, and 88 ms. The R2 map was calculated by fitting the exponential function *SI*(*t*) = *SI*_0_exp(−*t*/T2) to the signal intensity decay curve *SI*(*t*) given by the three TE images for each voxel of the T2-weighted sequence, whereas R2 is defined by R2 = 1/T2. The R2^*^ map was calculated in the same fashion using the three TE images of the T2^*^-weighted sequence. Finally, the R2′ map was calculated using the formula: 1/T2′ = 1/qT2^*^−1/qT2, whereas R2′ is defined by R2′ = 1/T2′. All computations required for R2/T2 mapping were performed using the in-house developed software tool Antonia (21).

The DTI sequence was acquired using a single-shot balanced echo-planar imaging sequence with TR = 4,500 ms, TE = 83 ms, and flip angle = 90°. A total of 27 slices with a thickness of 5 mm with an image in-plane resolution of 1.875 ×1.875 mm^2^ were acquired without diffusion gradients (b = 0 s/mm^2^) and with diffusion gradients (b = 1,000 s/mm^2^) applied along 20 non-collinear directions, averaged over two acquisitions. The DTI preprocess tool (22) was used for DTI processing to generate the diffusion parameter maps (mean diffusivity [MD], fractional anisotropy [FA], radial diffusivity [RD], and axial diffusivity [AD]).

### Image Registration

The automatic segmentation of anatomical brain regions was performed by registration of the Montreal Neurological Institute (MNI 152) brain atlas to each T1-weighted MPRAGE image using NiftyReg (23), which allows transforming atlas brain regions defined in the MNI space to each individual dataset for volumetric analysis. More precisely, the MNI brain atlas was registered to each T1-weighted MRI dataset using a rigid transformation followed by an affine transformation. The resulting affine transformation was then used for initialization of a non-linear registration using a cubic B-Spline parametrisation (free-form deformation). After this, the Harvard-Oxford cortical, Harvard-Oxford subcortical, MNI, and the Johns Hopkins University (JHU) white matter tractography atlas brain regions were transformed to each T1-weighted MRI dataset using the corresponding non-linear transformation and a nearest-neighbor interpolation. In addition to the parcellated atlas brain regions, the non-linear deformation field was also used for transforming a binary segmentation of the total intracranial volume to the T1-weighted dataset of each patient.

After this, the T2-weighted and diffusion-weighted datasets were non-linearly registered to the corresponding T1-weighted MRI dataset from the same patients using ANTs (24) to enable a combined analysis. More precisely, the average (b = 1,000 s/mm^2^) DTI image was used as the reference for this due to improved anatomical details and higher similarity to the T1-weighted dataset. This registration also consisted of a rigid followed by an affine transformation used for initialization of the non-linear registration, which was performed using a symmetric diffeomorphic image registration method, to allow correcting for distortion artifacts. The resulting transformation was then used to transform the DTI-derived parameter maps (MD, FA, RD, AD) to the corresponding T1-weighted MRI dataset from the same patient. The same approach was used for registration of the T2-weighted datasets to the T1-weighted MRI dataset whereas the dataset from the triple-echo T2-weighted sequence acquired with the longest echo time was used as the reference in this case. The resulting transformation was then used to transform the T2 parameter maps (R2, R2^*^, R2′) to the corresponding T1-weighted MRI dataset. All registration results were visually checked to ensure optimal registration quality, so that no missing values or significant outliers were present in the extracted features.

### Feature Extraction

An overview of the extracted features using the different brain atlas regions is presented in Table 2. In detail, the Harvard-Oxford subcortical atlas defined in the MNI reference space consists of 21 brain regions such as the thalamus, caudate, hippocampus, and brainstem. The Harvard-Oxford cortical atlas consists of 48 brain regions such as the insular cortex, precentral gyrus, and temporal pole. The MNI brain regions (not to be confused with the MNI 152 atlas used for registration) include nine well-known large structures such as the cerebellum, frontal lobe, temporal lobe, and others. Finally, the JHU atlas includes 20 structural white matter tracts such as anterior thalamic radiation, forceps major/minor, superior longitudinal fasciculus and others, which have been widely used in DTI-based studies.

**Table 2:** Overview of atlases and number of features extracted for regional analysis of morphology, brain iron accumulation/deposition, and microstructural integrity.

**Modality**	**Type of feature**	**Atlases used**	**Total number of extracted features**
T1-weighted MRI	**Morphology:** Regional Volume (V) Regional Brain Surface (SA) Regional Surface area to Volume Ratio (SA:V)	Harvard-Oxford Cortical	234
		Harvard-Oxford Sub Cortical	
		MNI brain regions	
T2-weighted MRI	**Brain iron content:** R2, R2*, R2′	Harvard-Oxford Cortical	396
		Harvard-Oxford Sub Cortical	
		Johns Hopkins University White Matter Tractography	
Diffusion-tensor MRI	**Microstructural integrity:** Mean diffusivity (MD) Fractional anisotropy (FA) Radial diffusivity (RD) Axial diffusivity (AD)	Harvard-Oxford Cortical	520
		Harvard-Oxford Sub Cortical	
		Johns Hopkins University White Matter Tractography	

In a first step, the registered Harvard-Oxford cortical, Harvard-Oxford sub-cortical, and MNI brain regions were used to quantify the volumes of the corresponding brain regions. To account for different global head sizes, the extracted volumes were normalized using the full intracranial volumes as described previously (25). Apart from the volume, the surface area as well as the surface-area-to-volume ratio (SA:V) was also determined for each brain region in the Harvard-Oxford cortical, Harvard-Oxford sub-cortical, and MNI brain atlas. For calculation of the SA:V, the raw regional volumes instead of the volumes corrected for the full intracranial volume were used since the SA:V metric is intrinsically normalized by dividing the surface area by the corresponding volume and would be skewed otherwise. Since the morphological parameters are of reduced interest for white matter tract analysis, the JHU atlas regions were not considered for morphological analysis. It should be noted that the regions used from the MNI brain atlas for the morphological analysis include the large lobes and cerebellum, which are composed of many of the smaller structures defined in the other atlases. These larger structures were included for the morphological analysis to quantify more global atrophy effects that might be missed when analyzing the small structures only. As each lobe contains a large number of the smaller structures defined in the other atlases, the measured morphological parameters for the large lobes are not likely to show high redundancy despite the overlap.

After this, the registered Harvard-Oxford cortical, Harvard-Oxford sub-cortical, and JHU atlas brain regions were used to quantify the corresponding median DTI parameters (MD, FA, RD, AD) and T2 parameters (R2, R2^*^, R2′) in the corresponding atlas brain regions. Median instead of average values were used to account for potential non-normal value distribution and partial volume effects at the border of brain structures. The MNI brain regions were not used for analysis of the DTI and T2-weighted datasets because the corresponding brain regions are rather large, so that localized microstructural and iron deposition differences would not be captured. Using this approach, a total of 1,150 features describing the regional morphology, iron accumulation/deposition, and microstructural integrity were extracted and used for training and testing of machine learning models described below.

### Machine Learning Pipeline

The machine learning framework implemented for this study consists of a feature ranking/selection and classification stage. In detail, features are initially ranked based on their relevance for the classification task using each of the following feature selection algorithms: correlation attribute evaluator, gain ratio/information gain evaluator, principle component analysis, RELIEFF, and support vector machine attribute evaluator. After this, the 100 highest ranked features for each ranking method were selected for initial inclusion for classification. This number was selected to decrease computational processing times associated with higher number of features but also to ensure that more observations than features are available to solve the classification problems. Each set of ranked features was then used to train each of the following classification models: simple decision tree, random forest, logistic model tree, k-nearest neighbors, naive Bayes, support vector machine (SVM), and multi-layer perceptron. All models were trained using the models implemented in Weka (26) with standard hyper-parameter values to reduce the risk of overfitting.

A leave-one-out cross validation routine was employed for classifier performance evaluation. To prevent double dipping, the leave-one-out cross validation also included the feature ranking. The optimal number of highest ranked features used for training and testing of the classifier was systematically optimized by iteratively removing the lowest ranked feature from the training and testing sets. This recursive feature elimination approach was performed by continuously removing the features from the initial 100 highest ranked features until only a single feature was left in the training and testing sets.

In order to identify the optimal combination of feature selection and classification method, the overall 3-level classification accuracy was used as the main evaluation metric. In case of equal classification performance, the feature selection and classification method combination requiring the lowest number of features is reported following the Occam's razor principle (27).

The pipeline described above was applied four times: (1) using the morphological features only, (2) using the R2 features only, (3) using the diffusion features only, and (4) using all features together.

## Results

In the following, the results of the best performing feature selection and classification combination using the single modality features and the combined multi-modal features, as well as the corresponding selected features are described.

### T1-Weighted MRI

Regional brain volume, surface area, and surface area to volume ratio features derived from structural T1-weighted MRI datasets resulted in a top overall accuracy of 65% differentiating PD, PSP-RS, and HC subjects, respectively. The confusion matrix and additional evaluation metrics for the optimal machine learning model are presented in Table 3. This accuracy was achieved when the top 40 features were selected using a combination of the gain ratio feature ranking and linear kernel SVM classification approach. In detail, the differentiation of HC subjects and patients with PD or PSP-RS was rather poor, with a total of 15 out of 38 misclassified HC subjects. Furthermore, classification of PD was also sub-optimal, resulting in 10 and seven PD patients wrongly classified as HC and PSP-RS, respectively. The top ranked features consisted mainly of sub-cortical structures such as brainstem, pallidum, putamen, thalamus, and cortical structures, including the occipital pole, frontal and temporal gyrus, and opercular gyrus. In addition, volume, surface area, and SA:V features were equally present in the ranking, indicating the importance of including various multi-aspect (not multi-modal) T1-weighted features and not only the simple volumes.

**Table 3:** Confusion matrix following a gain ratio + SVM classification combination using morphological features only.

**Morphology Features (Surface area, Volume, and Surface-Area-to-Volume Ratio Features)**											
**Class**	**TP Rate**	**FP Rate**	**Precision**	**Recall**	**F-Measure**	**MCC**	**ROC Area**	**Confusion Matrix**			**Accuracy**
								**HC**	**PD**	**PSP-RS**	
HC	0.605	0.185	0.657	0.605	0.630	0.429	0.710	23	12	3	65.0%
PD	0.622	0.241	0.667	0.622	0.644	0.384	0.690	10	28	7	
PSP-RS	0.800	0.120	0.615	0.800	0.696	0.619	0.840	2	2	16	

*TP, True Positive; FP, False Positive; MCC, Matthews Correlation Coefficient; ROC AUC, Area under the receiver operating characteristic curve; HC, Healthy Controls; PD, Parkinson's disease; PSP-RS, Progressive supranuclear palsy Richardson's syndrome*.

### T2-Weighted MRI

The classification results using the regional R2, R2^*^, and R2′ features are described in Table 4. In this case, the highest classification accuracy of 75.7% was obtained using the top 30 features selected by the principle component feature selection method in combination with a logistic model tree (LMT) classification model. In detail, nine HC subjects were misclassified as patients (7 PD and 2 PSP-RS), seven PD patients were wrongly classified as HC, and two PD patients wrongly classified as PSP-RS. In addition, five patients with PSP-RS were wrongly classified as PD. Overall, weighted combinations of brain iron metabolism measures in the cerebral cortex, left accumbens, left amygdala, and left hippocampus were present in the highest ranked principle component features.

**Table 4:** Confusion matrix following a PCA + LMT classification combination using brain iron content measures only.

**T2-weighted Image Features (based on quantitative R2, R2**^****′****^, and R2* **Features)**											
**Class**	**TP Rate**	**FP Rate**	**Precision**	**Recall**	**F-Measure**	**MCC**	**ROC Area**	**Confusion Matrix**			**Accuracy**
								**HC**	**PD**	**PSP-RS**	
HC	0.763	0.108	0.806	0.763	0.784	0.663	0.875	29	7	2	75.7%
PD	0.756	0.207	0.739	0.756	0.747	0.547	0.845	7	34	4	
PSP-RS	0.750	0.072	0.714	0.750	0.732	0.665	0.948	0	5	15	

*TP, True Positive; FP, False Positive; MCC, Matthews Correlation Coefficient; ROC AUC, Area under the receiver operating characteristic curve; HC, Healthy Controls; PD, Parkinson's disease; PSP-RS, Progressive supranuclear palsy Richardson's syndrome*.

### Diffusion-Tensor Imaging (DTI)

The classification results based on the DTI measurements alone (MD, FA, RD, AD maps) are shown in Table 5. The highest classification accuracy was obtained when the top 69 features were used. In detail, the information gain-based feature ranking method in combination with a logistic model tree (LMT) classification resulted in a classification accuracy of 95.1%. Overall, a total of five subjects were misclassified (three PD and two PSP-RS patients), whereas no HC subjects were misclassified. The highest-ranking diffusion features included mostly cortical regions such as the parahipocampal gyrus, cingulum, cingulate gyrus, opercular cortex as well as sub-cortical structures such as the thalamus, brainstem, and pallidum.

**Table 5:** Confusion matrix following an information gain + LMT classification combination using DTI maps only.

**Diffusion Tensor Imaging Features (MD, FA, RD, AD Features)**											
**Class**	**TP Rate**	**FP Rate**	**Precision**	**Recall**	**F-Measure**	**MCC**	**ROC Area**	**Confusion Matrix**			**Accuracy**
								**HC**	**PD**	**PSP-RS**	
HC	1.000	0.000	1.000	1.000	1.000	1.000	1.000	38	0	0	95.1%
PD	0.933	0.034	0.955	0.933	0.944	0.901	0.975	0	42	3	
PSP-RS	0.900	0.036	0.857	0.900	0.878	0.848	0.968	0	2	18	

*TP, True Positive; FP, False Positive; MCC, Matthews Correlation Coefficient; ROC AUC, Area under the receiver operating characteristic curve; HC, Healthy Controls; PD, Parkinson's disease; PSP-RS, Progressive supranuclear palsy Richardson's syndrome; MD, Mean diffusivity; FA, Fractional anisotropy; RD, Radial diffusivity; AD, Axial diffusivity*.

### Combining Multiple Modalities

The classification results based on the complete multi-modal feature set utilizing features from T1-weighted MRI (brain surface area, brain volume, SA:V), T2-weighted MRI (R2, R2^*^, and R2′), as well as DTI (MD, FA, RD, AD maps) are shown in Table 6. The highest classification accuracy in this multi-modal model was obtained when the top 79 features were used. In detail, the support vector machine feature ranking method in combination with a multi layer perceptron (MLP) classification resulted in the highest performance of 95%. Overall, a total of five subjects were misclassified including one PD and four PSP-RS patients, whereas none of the HC subjects were misclassified.

**Table 6:** Confusion matrix following an SVM based feature selection + MLP classification combination using features from multiple MRI modalities.

**Combination of All Imaging Features (Morphology, Brain Iron Content Marker, Diffusion)**											
**Class**	**TP Rate**	**FP Rate**	**Precision**	**Recall**	**F-Measure**	**MCC**	**ROC AUC**	**Confusion Matrix**			**Accuracy**
								**HC**	**PD**	**PSP-RS**	
HC	1.000	0.000	1.000	1.000	1.000	1.000	1.000	38	0	0	95.1%
PD	0.978	0.069	0.917	0.978	0.946	0.904	0.986	0	44	1	
PSP-RS	0.800	0.012	0.941	0.800	0.865	0.840	0.983	0	4	16	

*TP, True Positive; FP, False Positive; MCC, Matthews Correlation Coefficient; ROC AUC, Area under the receiver operating characteristic curve; HC Healthy Controls; PD, Parkinson's disease; PSP-RS, Progressive supranuclear palsy Richardson's syndrome*.

The selected features included features from all three MRI sequences (see Table 7). In detail, eleven features from the total set of 79 were morphological features, including deep gray matter regions such as the left pallidum, left and right thalamus, left caudate, and brainstem. Several cortical structures such as precentral and supramarginal gyrus, angular gyrus, temporal fusiform cortex, and planum polare were also among the top ranked features. Moreover, most of the morphological features selected for this classification problem included volume values rather than brain surface area or the SA:V. In terms of brain iron metabolism markers, a total of 12 features were present in the optimal feature set. Brain iron metabolism markers in areas such as the temporal fusiform, parahippocampal gyrus, frontal gyrus, and others were ranked highly according to the SVM-based feature selector with R2′ features being selected more than twice as often as R2 and R2^*^ features. All remaining top ranked features (*n* = 56) were diffusion features. Diffusion attributes in regions such as parahippocampal gyrus, insular cortex, pallidum, thalamus, brainstem, putamen, and others were ranked as the most discriminative features. Similar numbers of all diffusion parameters were selected.

**Table 7:** Feature composition of the SVM based feature selection + MLP classification combination using features from multiple MRI modalities.

**Modality**	**V**	**SA**	**SA:V**	**R2**	R2*	**R2^**′**^**	**FA**	**MD**	**AD**	**RD**	**Total**
T1-weighted	6	2	3	–	–	–	–	–	–	–	11
T2-weighted	–	–	–	2	3	7	–	–	–	–	12
DTI	–	–	–	–	–	–	15	12	13	16	56

*V, Volume; SA, Surface Area; SA:V, Surface-Area-to-Volume ratio; FA, Fractional Anisotropy; MD, Mean Diffusivity; AD, Axial Diffusivity; RD, Radial Diffusivity*.

## Discussion

Low accuracies of differential diagnosis between classical PD and PSP-RS based on established criteria, even when combined with standard anatomical MRI, have promoted the development of sophisticated machine learning frameworks for assisting with clinical diagnosis. Neuro-imaging data obtained from various MRI modalities have been widely employed for this purpose, whereas T1-, T2-, and diffusion-tensor MRI have been used most frequently. The main contribution of this work is that, for the first time, the benefit of a multi-parametric machine learning approach compared to uni-modal machine learning models was systematically investigated, whereas the results indicate that brain morphology and brain iron metabolism markers do not lead to quantitative benefits compared to using DTI features alone for an automatic classification of PD patients, PSP-RS patients, and HC subjects.

Overall, the results of this study are generally well in line with previous research as discussed in the following. However, it should be noted that it is not possible to directly compare the individual classification results of this study with others due to different patient samples, different evaluation schemes, and specific aims.

### T1-Weighted MRI

Most previously described classification models are based on morphological parameters determined using T1-weighted MRI datasets. While several other individual level classification studies using volumetric features have reported accuracies differentiating Parkinsonian syndromes ranging from <50% up to 86% [e.g., (14, 28)], previously described volumetric-based studies mostly report low accuracies differentiating patients with PD from HC subjects using volume-based features. The optimal morphology-based classifier in this study performed poorly classifying PD patients and HC subjects resulting in a ~26% error rate. Thus, the results obtained in this study for classifying PD patients and HC subjects using morphology features are consistent with previous studies. One potential reason for the poor ability of morphology metrics to differentiate PD patients and HC subjects could be related to less pronounced structural differences in these groups (29). Consequently, macro-structural features (i.e., morphology) do not seem sensitive enough to differentiate PD patients from HC subjects. In terms of PD vs. PSP classification, individual level classification methods using morphology features as, for example, previously described by Scherfler et al. (30), Sarica et al. (31), and Focke et al. (32) all reported comparably high classification accuracies. Unlike the PD vs. HC classification, it appears that features derived from high-resolution T1-weighted images are indeed valuable for differentiation of these two entities. Similarly, the optimal machine learning model in this work performed relatively well, only misclassifying 13% of the patients. Finally, studies focusing on PSP vs. HC classification have resulted in even higher accuracies of up to 94%. In detail, the highest accuracy for classification of PSP vs. HC with 93.98% was previously reported by Sarica et al. (31) by using volumetric features in a naive Bayes classifier validated by a 10-fold cross validation in a relatively large cohort of 46 HC, 65 PD, and 32 PSP. These results are to be expected considering the significant structural and functional differences in PSP patients compared to HC subjects. In the present study, a classification accuracy of 89% was achieved for the PSP-RS vs. HC classification in a three-level classification routine, also including HC subjects. Due to the clear potential for differentiating PSP-RS patients from PD patients and HC subjects, incorporation of morphological features within a computer-aided diagnosis framework appears still valuable and can add predictive value to such tools.

The main findings of this research project with implications to future developments of classification methods for PD vs. PSP-RS vs. HC differentiation using morphology features are 2-fold. First, the inclusion of the surface area and SA:V values provide complimentary information compared to using volumetric features only as the top features for this classification task consist (almost evenly) of a wide variety of all three morphological metrics, which is in line with findings from another recent study (25). Second, adding the morphological profiles of cortical structures for classification does not seem to improve the classification accuracy as most selected features belong to sub-cortical rather than cortical regions, which is in line with previous findings (25).

### T2-Weighted MRI

In a similar study, Boelmans et al. utilized brain iron metabolism markers in a logistic discriminant analysis approach to classify 24 HC, 30 PD, and 12 PSP subjects (16). Subsequently, the classification resulted in an overall accuracy of 74.2%. Using a similar classification pipeline, Eckert et al. achieved a total accuracy of 75.4% based on a sample of 20 HC, 15 PD, 10 PSP, and 12 multiple system atrophy (MSA) subjects (33). However, the two studies mentioned did not utilize a feature section method but employed a manual selection of a few brain regions. In this study, a similar accuracy of 75.7% was obtained using a sample size of 45 PD, 20 PSP-RS, and 38 HC subjects following an automatic PCA feature selection and LMT classification method. While the accuracies obtained are similar, there are three significant differences between the other two studies and ours. First, the patient sample used in this study is considerably larger. Second, the previous studies did not perform cross validation, whereas leave-one-out-cross-validation was used in this study. Third, the features used for classification in the two previous studies were handpicked and not automatically identified as done in this work, which could introduce a selection bias.

In terms of PD vs. HC classification, the optimal machine learning model identified in this study performs rather poorly with seven PD wrongly classified as HC subjects and vice versa. The misclassification in this category was also the main contributor for downgrading the overall classification performance. A potential explanation for this result could be that iron metabolism markers do not differ considerably between HC subjects and PD patients, therefore resulting in sub-optimal classification performance. Similar to morphological information, brain iron metabolism markers, seem to be inadequate features to differentiate between PD patients and HC subjects. Likewise, the differentiation of PSP-RS vs. PD patients was rather modest with four PD and five PSP-RS patients wrongly classified as PSP-RS and PD, respectively. While the optimal classifier using morphological features also resulted in a total of nine misclassified instances differentiating PD vs. PSP-RS, the difference is that misclassified patients using brain iron metabolism features were rather balanced between the two diseases, whereas morphological features exhibited a weaker PSP-RS classification ability. In addition, the differentiation of HC subjects vs. PSP-RS patients was far better resulting in only two PSP-RS subjects misclassified as HC. Thus, regional iron metabolism markers seem to differ considerably between PSP-RS patients and HC subjects, which is consistent with current literature (34).

Overall, brain iron metabolism markers do not seem to be good predictors for the differentiation of HC subjects from PD patients and PSP-RS from PD patients. Finally, it should be mentioned that the optimal feature selection method used for this classification task (PCA: principle component analysis) composes new features from a linear combination of the original features, which makes it more complicated to investigate how individual features from the initial feature set contribute to the overall classification. Generally, more research needs to be focused on investigating the association of the regional brain atrophy (i.e., morphological cell loss) and tissue iron accumulation.

### Diffusion-Tensor MRI

Various previous studies have shown that diffusion-based features can be used for a classification of PD patients and HC subjects. For example, Scherfler et al. (35), Salamanca et al. (36), and Banerjee et al. (37) used diffusion metrics such as MD (synonym: apparent diffusion coefficient [ADC]) and FA parameter maps for classification and obtained accuracies of up to 98%. This is generally in line with the findings of this work, with a top accuracy of 100% achieved for the classification of PD patients and HC subjects using an information gain feature ranking and LMT classification approach with a sample size of 45 PD patients and 38 HC subjects. Thus, it may be concluded that the differentiation potential of DTI measurements is much higher than the previously discussed regional brain morphological and iron metabolism features. One potential explanation for this finding is that micro-structural changes are assumed to occur earlier than macro-structural changes or measurable tissue iron accumulation (38).

With respect to differentiating PD and PSP-RS syndromes, two PSP-RS and three PD patients were wrongly classified as PD and PSP-RS, respectively. The obtained sub-syndrome classification accuracy is among the top results reported in literature thus far. However, it should be noted that this is not the first work to employ DTI measurements for classification of PD and PSP-RS subjects although previous research on this is rather scarce. For example, Haller et al. presented an approach to classify PD subjects (*n* = 17) and subjects with atypical forms (*n* = 23) of Parkinsonism using the RELIEFF feature selection method and a support vector machine classifier, and voxel-wise FA values as features (15). A correct classification between PD patients vs. patients with atypical forms was achieved in up to 97.5 ± 7.5%. However, it should be noted that the group of 23 subjects with atypical forms of Parkinsonism included only one patient with PSP while the other subjects in this group were, for example, diagnosed with MSA, dementia with Lewy bodies, vascular Parkinsonism, and even traumatic brain injury. Thus, the results are not really comparable to those described in this work.

Several interesting conclusions can be made by investigating the results of the optimal classification model and selected features in more detail. First, it appears that diffusion changes are a global effect since cortical as well as sub-cortical structures brain regions can be found among the highest ranked features selected for the classification. The 69 selected features used for classification included brain regions that are well-known to be affected in by PD such as the brainstem, and deep gray matter structures including the thalamus, putamen, and pallidum. Additionally, brain regions that are part of the frontal cortex, namely the superior frontal gyrus and frontal medial cortex are among the selected regions, which are part of the prefrontal dopaminergic system. Additionally, it appears useful to investigate all four diffusion parameters as similar numbers of the four DTI features were selected for the optimal machine learning model. Within this context, it should be noted that most previous studies have only used FA and MD values for classification or group-wise studies. Similar to the different morphological features, the incorporation of multi-facet DTI features seems to have the potential to improve machine learning models by elucidating several aspects of PD syndromes.

### Multi-Modal Features

Several studies have been performed in the past aiming to classify patients with PD and HC patients by combining multi-modal image features. For example, Long et al. utilized structural and functional MRI information to classify PD patients and HC subjects (17). The results reported show that the combination of features leads to an improved classification accuracy of 86.9%. In a study similar to this work, Peran et al. combined T1-weighted, T2-weighted, and diffusion-weighted MRI features and reported a PD vs. HC classification accuracy of 95% for the corresponding machine learning model (18). In line with these findings, the optimal PD vs. HC classification model developed in this work using the multi-modal MRI data achieved a slightly better accuracy of 100% for differentiating PD patients and HC subjects. However, unlike the aforementioned studies that performed binary classifications, the differentiation task in this study was inherently more complex as three classes (PD, PSP-RS, HC) were considered. While the differentiation of PD vs. HC using the full multi-modal feature set outperformed single modality machine learning models using morphological and iron content markers alone, it was not superior compared to the machine learning model using only DTI features. In line with this finding, most of the features selected for the final model were DTI parameters.

Similarly, multiple studies have also attempted to develop and evaluate multi-modal machine learning models for classification of PD vs. PSP. For example, Morisi et al. combined volumetric, diffusion, and proton spectroscopy measures within a linear kernel SVM model and obtained an accuracy of 98% (39). Cherubini et al. used volumetric and diffusion features from a large sample of 57 PD and 21 PSP patients and achieved an accuracy of 100% using only white matter features (40). In the present study, a PSP vs. PD differentiation of 93% was achieved using multi-modal MRI features, which is in the range of the previously described results of binary classification problems. While the multi-modal machine learning model clearly outperforms the corresponding machine learning models using morphology or brain iron accumulation features alone, the accuracy is on par with the machine learning model using DTI parameters only. However, the multi-modal model was more precise correctly classifying PD patients, but less precise correctly classifying PSP patients compared to the machine learning model using DTI metrics only.

### Classification Model

A large number of feature selection methods such as information gain, principal component analysis, linear-kernel SVM based feature selection, RELIEFF, Fisher vector algorithm, evolutionary based techniques, fuzzy based data transformation, graph theory methods, and others have been employed in previous studies. At the same time, many previous studies did not incorporate any feature selection algorithm and simply employed pre-selected sub-syndrome specific features from a limited number of regions-of-interest instead. Within this context, the use of feature selection methods allows identifying important regions in a data-driven way, while manually selecting brain regions for a machine learning model might miss important regions.

Similar to the feature selection methods, a wide range of classification techniques such as support vector machines, different types of decision trees, linear and logistic regression, multi-layer perceptron models, and others have been used in previous machine learning models.

The fact that different setups of feature selection methods and machine learning models were found to be optimal for the different classification tasks in this work shows that it is important to test and evaluate different setups to identify the optimal setup for a given classification task. Within this context, it is important to ensure that the developed models do not overfit the training data. For this reason, the machine learning models were trained and tested only using the standard hyper parameters in this work. Additionally, the results of all machine learning models were carefully checked and none of the models reported outperformed the next best machine learning model by far. Thus, further classification improvements might be possible by further fine-tuning of the hyper-parameters. Nevertheless, we are confident that the main findings of this research study will still hold true.

### Limitations

This study has some limitations that should be discussed. First, the study cohort used in this work, while relatively large compared to similar studies, is still not large enough to fully expand on the generalizability of the proposed model. This limitation is further perpetuated by the lower incidences for PSP-RS compared to PD. Furthermore, an independent validation dataset, preferably acquired in different imaging centers, would be a more rigorous approach of model verification. However, this separate dataset was not available for this present study to further test the proposed model. We opted not to separate the current dataset into completely separate training and validation sub-groups as the training cohort would not have been sufficiently large enough to train a generalized classifier, potentially resulting in an over-fitted model. It is worth noting that studies employing separate validation datasets are rather scarce in this context, so that cross validation methods are used most frequently for classifier validation. Second, the ground truth classifications were determined by an expert clinician according to established consensus criteria without neuropathological proofs. Thus, there may be still a minor level of uncertainty left regarding the ground truth classification used for training and evaluation of the classifier. It should also be mentioned that due to the retrospective design of this study, the ground truth diagnoses were not based on the most recent guidelines for PD and PSP-RS diagnosis (41–43). However, all patients and participants included in this study were seen by two movement disorder specialists at least two times ensuring highly probable ground truth classifications. Third, the patient groups differed considerably regarding the age and sex distribution, which might bias the results obtained in this research project towards higher accuracies. Ideally, datasets from subjects with similar age and sex distributions should be used for training and testing to obtain more accurate and generalizable classification models. However, this is not an easy task as obtaining data with such strict specifications is time-consuming and expensive and is an undertaking that was not feasible for this research project due to the retrospective data analysis. Finally, it should be mentioned that only conventional machine learning techniques were used in this study instead of sophisticated deep learning models, which have recently shown good achievements for PD classification (44). However, the machine learning models used in this work require less data compared to novel deep learning models and, thus, allowed investigating different imaging setups in detail given the sample size available. We believe that the main findings of this study also hold true for novel deep learning methods with a deep learning model using DTI parameter maps performing on par or outperforming deep learning models based on T1-weighted datasets or T2-weighted datasets alone or a combination of all of these images. Nevertheless, this needs to be investigated in future studies with larger sample sizes as deep learning models are assumed to be more “data-hungry” compared to traditional machine learning techniques.

## Conclusion

The results of this study suggest that machine learning models using regional brain microstructural integrity metrics computed from DTI datasets perform similar to multi-modal machine learning models incorporating additional imaging metrics such as brain morphology and iron metabolism to differentiate PD, PSP-RS, and HC subjects.

## Data Availability Statement

The data analyzed in this study is subject to the following licenses/restrictions: The datasets generated during and/or analyzed during the current study are not publicly available due to containing information that could compromise the privacy of research participants but are available upon reasonable request with approval of the local ethics board. Requests to access these datasets should be directed to nils.forkert@ucalgary.ca.

## Author Contributions

AT: study design, image processing, data analysis, and drafting the manuscript and revising it critically. JS: data acquisition and critical revision of the manuscript. KB: study design, data acquisition, and critical revision of the manuscript. NF: study design, image processing, data analysis, and drafting the manuscript and revising it critically. All authors contributed to the article and approved the submitted version.

## Conflict of Interest

NF owns equity in Eppdata GmbH. The remaining authors declare that the research was conducted in the absence of any commercial or financial relationships that could be construed as a potential conflict of interest.
